# Lineage-Specific Chimerism and Outcome After Hematopoietic Stem Cell Transplantation for DOCK8 Deficiency

**DOI:** 10.1007/s10875-021-01069-5

**Published:** 2021-06-02

**Authors:** Johannes Raedler, Thomas Magg, Meino Rohlfs, Christoph Klein, Tanja Vallée, Fabian Hauck, Michael H. Albert

**Affiliations:** 1 Department of Pediatrics, Dr. Von Hauner Children’s Hospital, University Hospital, Ludwig-Maximilians-Universität München, Munich, Germany; 2 Munich Centre for Rare Diseases (M-ZSELMU), University Hospital, Ludwig-Maximilians-Universität München, Munich, Germany

**Keywords:** DOCK8 deficiency, HSCT, Mixed chimerism

## Abstract

Bi-allelic variants in the dedicator of cytokinesis 8 (*DOCK8*) gene cause a combined immunodeficiency, characterized by recurrent sinopulmonary and skin infections, food allergies, eczema, eosinophilia, and elevated IgE. Long-term outcome is poor given susceptibility to infections, malignancy, and vascular complications. Allogeneic hematopoietic stem cell transplantation is currently the only curative treatment option and has shown promising outcome. The impact of mixed chimerism on long-term outcome is unclear. We reasoned that reversal of disease phenotype would depend on cell lineage-specific chimerism. *DOCK8* variants were confirmed by Sanger and/or exome sequencing and immunoblot and/or intracellular flow cytometry. Donor chimerism was analyzed by XY-fluorescence in situ hybridization or quantitative short tandem repeat PCR. Outcome was assessed by laboratory tests, lymphocyte subsets, intracellular DOCK8 protein flow cytometry, T-cell proliferation analysis, and multiparameter immunoblot allergy screening. We report on nine patients, four of whom with mixed chimerism, with a median follow-up of 78 months after transplantation. Overall, we report successful transplantation with improvement of susceptibility to infections and allergies, and resolution of eczema in all patients. Immunological outcome in patients with mixed chimerism suggests a selective advantage for wild-type donor T-cells but lower donor B-cell chimerism possibly results in a tendency to hypogammaglobulinemia. No increased infectious and allergic complications were associated with mixed chimerism. Aware of the relatively small cohort size, we could not demonstrate a consistent detrimental effect of mixed chimerism on clinical outcomes. We nevertheless advocate aiming for complete donor chimerism in treating DOCK8 deficiency, but recommend reduced toxicity conditioning.

**Supplementary Information:**

The online version contains supplementary material available at 10.1007/s10875-021-01069-5.

## Introduction

The inborn error of immunity (IEI) caused by bi-allelic dedicator of cytokinesis 8 (*DOCK8*) deficiency was first described in 2009 by Zhang et al. [1]. The resulting combined T- and B-cell immunodeficiency in DOCK8 deficiency presents with food allergies, eczema, eosinophilia, and elevated IgE, explaining why it was historically categorized as an autosomal-recessive hyper-IgE syndrome (HIES) [2–4]. Recurrent sinopulmonary infections with bronchiectasis, skin infections, lymphopenia, and hypogammaglobulinemia are other frequent findings [2, 3, 5]. Common infectious agents are bacteria and fungi, DNA viruses, or molluscum contagiosum. A T_H_1-deficient phenotype and cytokine abnormalities with T_H_2 activation may explain eosinophilia and IgE overproduction in DOCK8 deficiency [6, 7]. Diagnosis may be delayed or missed in patients with milder or varying phenotypes or near-normal flow cytometric DOCK8 expression caused by missense mutations or somatic reversions [3, 5, 8, 9]. The long-term natural disease outcome is poor, given infectious or vascular complications and an increased susceptibility to malignancy, predominantly of hematological or epithelial origin. Median survival is less than 20 years and almost all patients experience a life-threatening complication by the age of 25, despite prophylactic treatment [10].

DOCK8 is an atypical guanine nucleotide exchange factor of the DOCK 180 superfamily, which interact with Rho GTPases regulating cytoskeletal rearrangements [1, 5, 10, 11], partially explaining phenotypical overlap with other IEIs caused by cytoskeletal dysregulation, especially Wiskott-Aldrich syndrome (WAS) and CARMIL2 deficiency [12, 13]. Interaction between DOCK8 and WASP in NK-cells has been reported [2, 14, 15]. In comparison, the IEI caused by STAT3 deficiency, autosomal-dominant HIES, presents with different phenotypical features, such as connective tissue, dental, and skeletal abnormalities, and typically exhibits a less severe immunodeficiency [2, 3, 5, 16–18].

Previous reports suggest that allogeneic hematopoietic stem cell transplantation (HSCT) offers a curative therapeutic option for DOCK8 deficiency [2, 12, 19–22]. A recent retrospective analysis of a large cohort confirmed favorable outcomes after HSCT and suggested good safety and efficacy of reduced toxicity conditioning regimens. A detailed analysis of donor chimerism and immunological reconstitution was however not possible in this multi-center retrospective analysis [23]. Treosulfan or reduced-dose busulfan-based reduced toxicity regimens offer less short-term and possibly long-term toxicity [24]. However, they do not always result in complete donor chimerism of all cell lineages, which—depending on the underlying condition—may or may not result in reversal of the disease phenotype [25]. For example, in familial hemophagocytic lymphohistiocytosis, a donor T-cell chimerism of about 20–30% is deemed sufficient [26], regardless of the chimerism in other cell lineages, while in WAS complete donor chimerism is necessary for full disease correction [27]. Reports on immunological outcome of DOCK8 patients after HSCT are relatively scarce, especially for patients with mixed chimerism [19, 28, 29].

Given the fact that DOCK8 deficiency affects many hematopoietic lineages, we reasoned that reversal of disease phenotype after HSCT would depend on the level of cell lineage-specific donor chimerism. We here report detailed analysis of long-term clinical outcome, lineage-specific chimerism, laboratory parameters, and pulmonary function tests of nine patients who underwent HSCT for DOCK8 deficiency between 2004 and 2017, four of whom with ensuing variable degrees of mixed chimerism.

## Methods

### Data Acquisition

Data were retrieved from archived patients’ files. All laboratory analyses were part of the regular long-term follow-up schedule. All patients or their caregivers consented to scientific analysis of their data. P4 was lost to follow-up after 2.5 years. All patients have been part of previous publications [2, 20, 23].

### Flow Cytometry

Flow cytometry for T-, B-, and NK-cell subsets was performed as described [13]. DOCK8 intracellular staining flow cytometry was performed using Cytofix/Cytoperm buffer set (Becton Dickinson, San Jose, USA (BD)), anti-DOCK8 (G-2, 1:20, Santa Cruz, Dallas, USA), and mouse FITC-conjugated secondary antibody (RMG1-1, 1:200, BioLegend, San Diego, USA) followed by blocking with normal mouse IgG (Thermo Fisher Scientific, Waltham, USA) and surface staining with PE-anti-CD3 (SK7, 1:10, BD), PC7-anti-CD19 (J3-119, 1:50, Beckman Coulter, Brea, USA), and APC-anti-CD56 (NCAM16.2, 1:50, BD).

### Immunoblot Analysis

For immunoblot analysis, whole cell lysates were prepared from T-lymphoblasts as described [13]. Total protein was resolved on SDS-PAGE, transferred to nitrocellulose membrane, and probed with anti-DOCK8 (G-2, 1:1000), anti-GAPDH (6C5, 1:3000), and goat anti-mouse IgG-HRP (sc-2005, 1:10,000, all Santa Cruz).

### Proliferation

PBMC were labeled with 2.5 µM carboxyfluorescein succinimidyl ester (CFSE, Thermo Fisher Scientific, Waltham, MA) and stimulated with anti-CD3-coupled beads (anti-Biotin MACSiBeads, Miltenyi Biotec, Bergisch Gladbach, Germany, coupled with Biotin-anti-CD3, OKT3) at a ratio of 5:1 with and without 1 µg/ml CD28 (CD28.2, both Thermo Fisher Scientific) or with 0.5 ng/ml PMA and 1 µM ionomycin (Sigma-Aldrich, St. Louis, MO). T-cell proliferation was measured by labeling PBMC with APC-H7-anti-CD3 (SK7, 1:50), APC-anti-CD4 (SK3, 1:50), PacB-anti-CD8 (RPA-T8, 1:50), and PE-anti-CD25 (M-A251, 1:25, all BD) 5 days after stimulation as described [13].

### Exome and Sanger Sequencing

Exome sequencing was performed at the Dr. von Hauner Children’s Hospital NGS facility as previously described [13]. Briefly, genomic DNA from whole blood was used for preparation of whole-exome libraries using the SureSelect XT Human All Exon V6 + UTR kit (Agilent Technologies, Ratingen, Germany) and subsequently sequenced with a NextSeq 500 platform (Illumina, San Diego, CA) to an average coverage depth of 90 × . Bioinformatic analysis used Burrows-Wheeler Aligner (BWA 0.7.15), Genome Analysis ToolKit (GATK 3.6), and Variant Effect Predictor (VEP 89). Frequency filtering was done against public (e.g., GnomAD, ExAC, and GME) and in-house databases. Potentially causative variants were confirmed by Sanger sequencing.

### Laboratory and Statistical Analyses

Donor chimerism for opposite-sex transplantations was analyzed by XY-fluorescence in situ hybridization (FISH), and by quantitative short tandem repeat PCR for same-sex transplantations, after MACS-based cell sorting, respectively. Allergy screening was performed by multiparameter immunoblots (Euroimmune, Lübeck, Germany).

Statistical analysis was performed with Prism version 7.0 (GraphPad, La Jolla, CA) using unpaired two-tailed T-tests with Welch’s correction not assuming equal variances, accepting *P* < 0.05 as significant. Central tendencies are reported as median values. Mean values are provided for selected results.

## Results

### Patients and HSCT

**Fig. 1:**
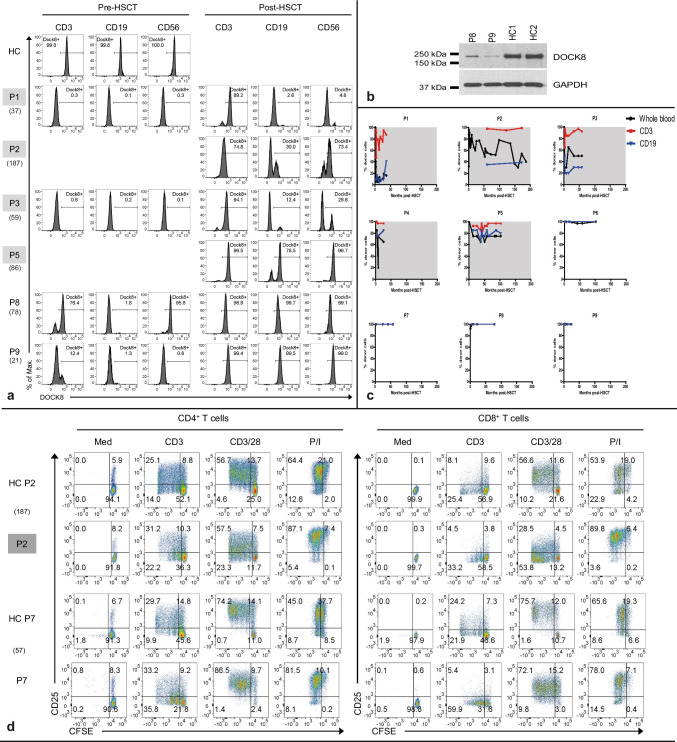
Complete donor chimerism results in complete expression of DOCK8 post-HSCT. **a** Flow cytometric analysis of intracellular DOCK8 expression for select patients at given time points and healthy control (HC) in CD3^+^ T-cells, CD19^+^ B-cells, or CD56^+^ NK-cells pre- and post-HSCT. FISH- or PCR-based detection of mixed chimerism (gray background) correlates with incomplete flow cytometric DOCK8 expression. P8 and P9 showed residual DOCK8 expression pre-HSCT due to hypomorphic mutations and somatic reversion, **b** confirmed by immunoblot analysis of DOCK8 expression in T-lymphoblasts of P8 and P9 pre-HSCT compared to HCs. **c** PB donor chimerism is shown for each patient in months post-HSCT as whole blood or lymphocyte subsets of CD3^+^ or CD19^+^ cells. A selective advantage for CD3^+^ donor T-cells is noted for patients with mixed chimerism. **d** Representative pseudocolor plots of CD25 surface expression and CFSE-dilution on CD4^+^ and CD8^+^ T-cells without (Med) and after stimulation for 5 days with anti-CD3, anti-CD3/CD28, or PMA/ionomycin (P/I) stimulation for P2 and P7 at given time points post-HSCT compared to respective HC

This report comprises nine patients with DOCK8 deficiency, confirmed by bi-allelic *DOCK8* variants by Sanger and/or exome sequencing, who received allogeneic HSCT after reduced toxicity conditioning at our institution between 2004 and 2017 (Table 1). P8 and P9 exhibited mosaic DOCK8 expression in CD3^+^, CD19^+^, and CD56^+^ cells compatible with somatic reversions (Fig. 1a and b). P1 suffered from various co-morbidities before HSCT, including a corrected cardiac defect (Shone complex), corrected cleft palate with residual dysphagia and seizures, with resulting dystrophia and delayed psychomotor development. P2 was the first patient ever reported with successful HSCT for DOCK8 deficiency; however, transplantation in 2004 preceded the first description of the underlying genetic cause [20]. HSCT details are presented in Table 1. Notable long-term morbidity was a presumably total-body irradiation (TBI)-related thyroid cancer with pulmonary metastasis in P2 treated surgically with ongoing remission. We report on a cumulative follow-up of 727 patient months post-HSCT and a median follow-up of 78 months (33–187).

**Table 1:**
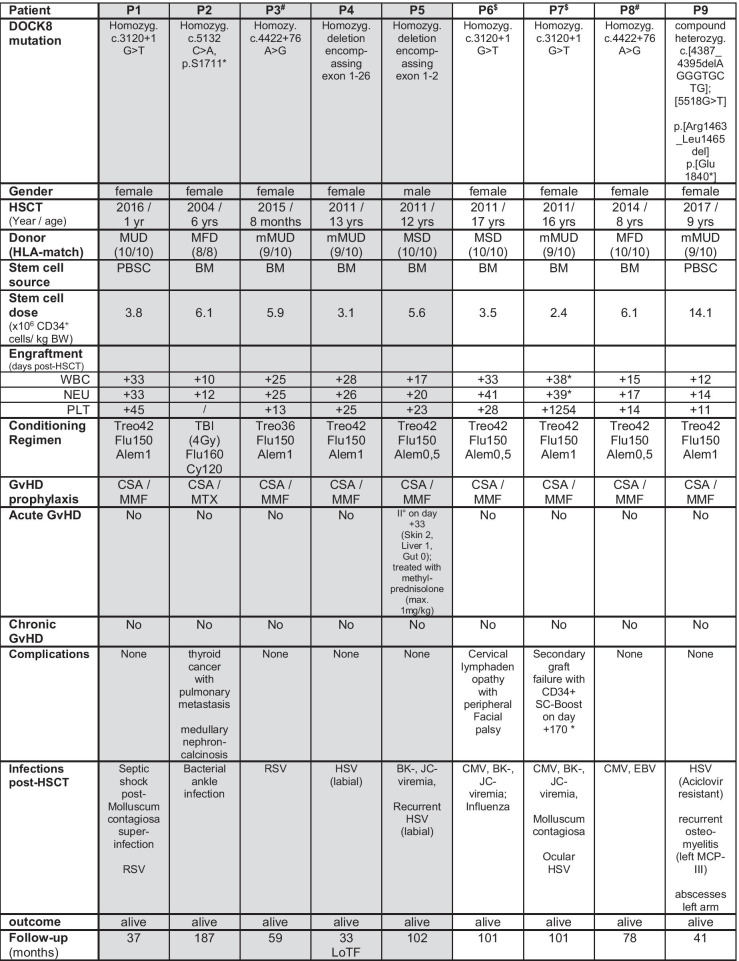
Patient and HSCT characteristics

Gray background highlights patients with mixed chimerism. *Alem* alemtuzumab (in mg/kg), *BM* bone marrow, *BW* body weight, *CSA* cyclosporine, *Cy* cyclophosphamide (in mg/kg), *Flu* fludarabine (in mg/m^2^), *HLA* human leucocyte antigen, *LoTF* loss to follow-up, *mMUD* mismatched unrelated donor, *MRD* matched related donor, *MUD* matched unrelated donor, *MTX* methotrexate, *NEU* neutrophils, *PBSC* peripheral blood stem cells, *PLT* platelets, *TBI* total-body irradiation, *Treo* treosulfan (in g/m^2^), *WBC* white blood cells. § and # denote siblings; “*” (asterisk) = P7 received a CD34 + stem cell boost on day + 170; “/” (slash) = no thrombocytopenia < 50.000/µl for P2 during HSCT

### Hematologic and Immunologic Reconstitution

Complete donor chimerism was present in four patients (P6–P9; Fig. 1a and c, Table 2). Kinetics of lineage-specific peripheral blood chimerism are shown in Fig. 1c. In patients with mixed chimerism, median whole blood donor chimerism was 50% (16–75%), whereas median donor CD3^+^ T-cell chimerism was 97% (range: 87–99%) and median CD19^+^ B-cell chimerism was 41% (30–85%). Post-HSCT DOCK8 expression by flow cytometry was assessed for six patients and corresponded well with lineage-specific chimerism measured by XY-FISH or PCR (Fig. 1a and c, Table 2). We also measured CD16^+^CD56^+^ NK-cell chimerism by flow cytometry, revealing a median of 52% (5–99%), seemingly independent from the degree of T-cell chimerism.

**Table 2:**
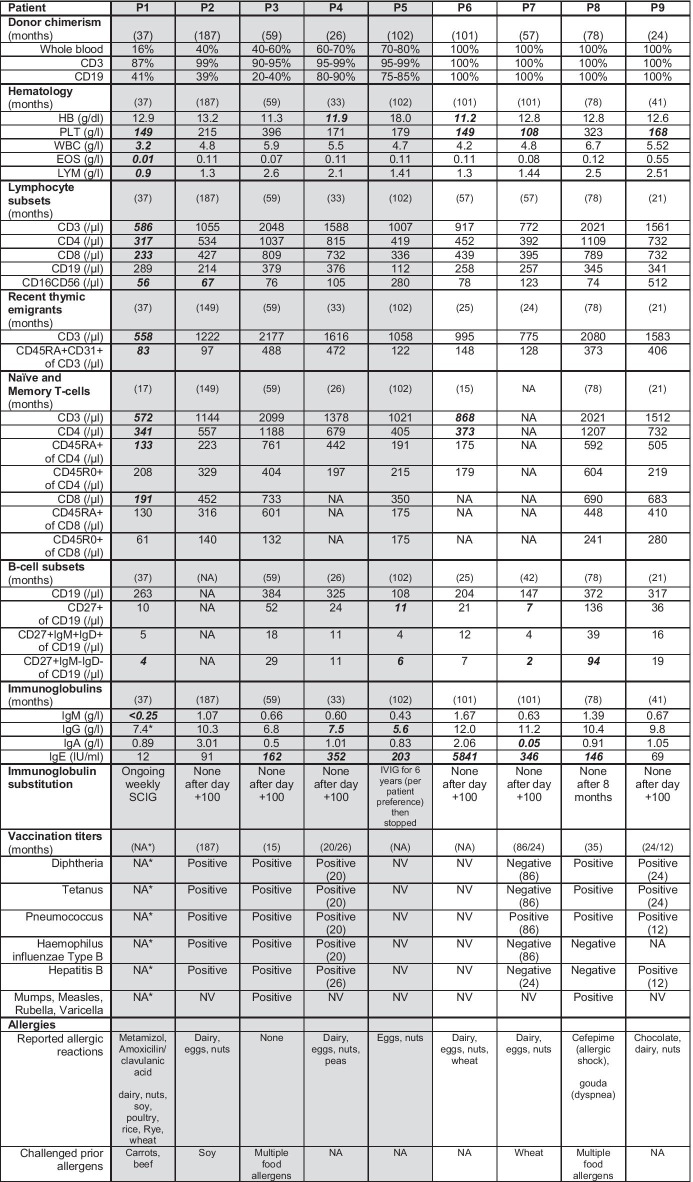
Outcome post-HSCT

Most recent results for donor chimerism, blood count, lymphocyte subpopulations, immunoglobulin levels, and vaccination titers are shown. Reported allergies and challenged prior allergens are subject to patient or guardian reports. Time of analysis is given in months post-HSCT. Gray background highlights patients with mixed chimerism. Bold denotes values outside age-adjusted norm

*EOS* eosinophils, *HB* hemoglobin, *IVIG* intravenous immunoglobulin substitution, *LoTF* loss to follow-up, *LYM* lymphocytes, *NA* not available, *NV* no vaccination performed, *PLT* platelets, *RTE* recent thymic emigrants, *SCIG* subcutaneous immunogloblin substitution, *WBC* white blood cells. “*” (asterisk) = weekly subcutaneous immunoglobulin substitution

No patient experienced primary graft failure (Table 1). P7 received a CD34^+^ stem cell boost from the original donor on day + 170 for poor graft function following CMV-, BK-, and JC-viremia and ganciclovir treatment. At last follow-up, all patients had (near) complete hematologic reconstitution with no significant differences between patients with mixed or complete donor chimerism. Median hemoglobin value was 12.8 g/dl (mixed chimerism: 12.9, complete donor chimerism: 12.7; *P* = 0.42) with mild anemia in P4 and P6 (P6 has iron deficiency anemia). Median platelet count was 171 g/l (mixed: 179, complete: 159; *P* = 0.61) with mild thrombocytopenia in four patients and median leucocyte count was 4.8 g/l (mixed: 4.8, complete: 5.2; P0.52) with mild lymphopenia in P1, whose Shone complex was corrected by cardiac surgery with partial thymectomy pre-HSCT (Table 2).

Complete immunologic reconstitution as defined by normal values for lymphocyte subsets was seen in six patients with no significant differences between patients with mixed or complete donor chimerism (Table 2). Median cell counts were 1055/µl CD3^+^ T-cells (mixed: 1055, complete: 1239; *P* = 0.88), 534/µl CD4^+^ helper T-cells (mixed: 534, complete: 592; *P* = 0.83), and 439/µl CD8^+^ cytotoxic T-cells (mixed: 427, complete: 586; *P* = 0.61). Moderate CD4^+^- and severe CD8^+^-T-lymphopenia were present in P1. Median CD19^+^ B-cell count was 289/µl (mixed: 289, complete: 300; *P* = 0.66). Median CD16^+^CD56^+^ NK-cell count was 78/µl (mixed: 76, complete: 101; *P* = 0.52) with mild NK-lymphopenia in P1 and P2. Kinetics of immunologic reconstitution are shown in figure S1.

Thymopoiesis, as assessed by recent thymic emigrants (RTE; CD3^+^CD45RA^+^CD31^+^), was reduced in P1 (after thymectomy), with overall median RTE counts of 148/µl (mixed: 122, complete: 261; *P* = 0.93; Table 2). Naïve (CD45RA^+^) and memory (CD45R0^+^) T-cell subsets were available for eight patients for CD4^+^ and six patients for CD8^+^ T-cell-subsets, showing a reduction in memory CD8^+^ cells in patients with mixed chimerism (median overall: 158/µl, mixed: 136, complete: 261; *P* = 0.016). However, this may be skewed by partial thymectomy in P1, limited data availability, and biased ratios of naïve versus memory T-cells in P3 post-HSCT. T-cell proliferation to CD3/CD28 stimulation was normal in seven patients (Fig. 1d), missing for P4 and P9, who had normal T-cell proliferation to antigen stimulation (tetanus and diphtheria; data not shown). P6 had a pathologic reaction to antigen stimulation, but she was not vaccinated, maybe accounting for that. P8 has a reduced proliferation to candida stimulation (not shown).

Analysis of B-cell subsets was available for eight patients (Table 2), showing no significant differences between patients with complete or mixed chimerism. Median CD27^+^-memory-B-cell count was 27/µl (mixed: 18, complete: 29; *P* = 0.45), median IgM-memory-B-cells (CD19^+^CD27^+^IgD^+^IgM^+^) count was 12/µl (mixed: 8, complete: 14; *P* = 0.37), and median switched-memory-B-cell (CD19^+^CD27 + IgD^−^IgM^−^) count was 9/µl (mixed: 9, complete: 13; *P* = 0.29). A reduction in switched-memory-B cells was observed in three patients. Hereof, P7 had a complete donor chimerism and P1 and P5 had mixed chimerism. P1, P4, and P5 present with persistent hypogammaglobinemia at last follow-up, defined as IgG values below the age-adjusted normal range (Table 2). P1 receives weekly subcutaneous immunoglobulin (SCIG) substitution. P5 received intravenous immunoglobulin (IVIG) substitution every 4 to 8 weeks per patient preference, but discontinued substitution after 6 years without subsequent infectious complications. Excluding P1, median serum IgG level was significantly reduced in patients with mixed chimerism (overall: 10.1 g/l, mixed: 7,7, complete: 10.8; *P* = 0.047). IgG kinetics post-HSCT are shown in figure S2; no IgG-subclass analyses were performed. Overall median serum IgM level was 0.64 g/l including P1 (mixed: 0.60, complete: 1.03, *P* = 0.16) and median serum IgA level was 0.91 g/l (mixed: 0.89, complete: 0.98; *P* = 0.67). Pre-HSCT IgG levels were not analyzed because all patients except P4 were on Ig substitution.

Production of specific antibodies in response to vaccinations is shown in Table 2. Two patients were not vaccinated post-HSCT. P7, who also has reduced switched-memory-B-cells, generally showed poor humoral responses to vaccinations. Vaccination with lived-attenuated viruses (mumps, measles, rubella, varicella) was declined by seven patients, but serological testing suggests protective titers in those vaccinated.

In summary, these results show satisfactory immune reconstitution post-HSCT in all patients except P1, who had partial thymectomy pre-HSCT. A tendency to hypogammaglobulinemia in patients with mixed chimerism was observed. No further significant correlation between mixed chimerism and incomplete hematologic or immunologic reconstitution could be detected in this cohort.

### Infections

Viremia was frequently diagnosed during pharmacological immunosuppression post-HSCT and treated with virostatic pharmacotherapy (Table 1). Notably, P9 experienced an aciclovir-resistant HSV infection (genetically proven) post-HSCT, which was successfully treated with foscarnet. At last follow-up, only P8 reported continued susceptibility to infection. She has recurrent respiratory infections presumably on the grounds of advanced structural lung damage pre-HSCT and requires regular prophylactic antibiotic treatment. P1 is free from severe infections under immunoglobulin replacement. Further noteworthy infections post-HSCT are shown in Table 1. Overall, infection frequency notably decreased after more than 1 year post-HSCT. No correlation between infectious complications and mixed chimerism was apparent.

### Allergies

**Fig. 2:**
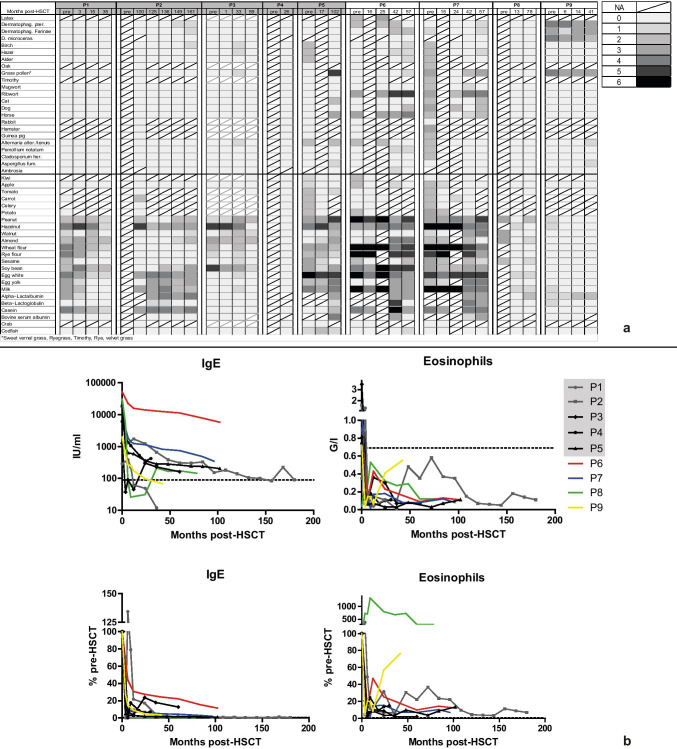
Mixed chimerism does not result in increased allergic diathesis post-HSCT. **a** Semi-quantitative immunoblot (Euroimmune, Lübeck, Germany) reactivity to specific allergens is shown at given time points (0 = no reactivity, 6 = strong reactivity) with food allergens below the horizontal line. **b** Course of serum IgE (IU/ml) and eosinophil count (G/l) pre- (time = 0) and post-HSCT. Lower panels show these as percentages of pre-HSCT values. Dashed lines define upper normal range. Gray background denotes patients with mixed chimerism

Eczema or atopic dermatitis resolved quickly post-HSCT in all patients and the allergic diathesis abated substantially. Two patients reported no allergies at last follow-up and the remainder reported mainly food allergies, specifically to dairy, egg, nut, or wheat products (Table 2). P8 reported severe allergic reactions to Gouda cheese and cefepime. In general, reported allergies post-HSCT were consistent with pre-HSCT conditions. Challenges of few food allergens showed tolerance post-HSCT, but exposure was mainly avoided per patient preference. Semi-quantitative multiparameter immunoblots were available for six patients for pre- and post-HSCT comparison (Fig. 2a), showing no obvious correlation between allergic tendency on immunoblot and donor chimerism. Skin prick testing was not performed. Overall, patients with high reactivity to food allergens on immunoblot pre-HSCT retained this post-HSCT without good correlation with clinical symptoms.

Total serum IgE was slowly down-trending post-HSCT (median IgE 17,438 IU/ml pre-HSCT), but continues to be elevated in five patients with a median level of 203 IU/ml (mixed: 203, complete: 246; *P* = 0.39). Eosinophilia resolved post-HSCT with a median eosinophil count of 0.11G/l (mixed: 0.11, complete: 0.12; *P* = 0.32), compared to 0.82G/l pre-HSCT (Fig. 2b, Table 2).

**Fig. 3:**
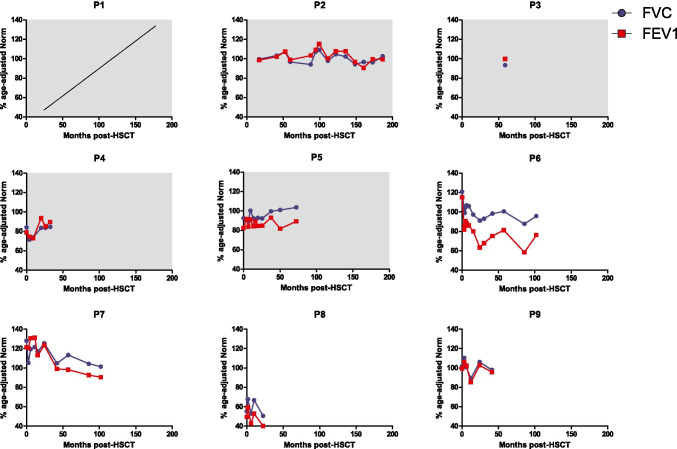
Mixed chimerism does not correlate with persistent airway obstruction post-HSCT. Spirometric results for FVC and FEV1 as percentages of age-adjusted normal values. Airway obstruction was defined as FEV_1_/FVC < lower limit, with light obstruction FEV_1_ > 60% age-adjusted norm, moderate obstruction FEV_1_ 30–60% age-adjusted norm, and severe obstruction FEV_1_ < 30% age-adjusted norm. P1 is too young for spirometric testing. Gray background denotes patients with mixed chimerism

Bronchial hyperreactivity to aerosol allergens was documented in five patients pre-HSCT (P2, P5, P6, P7, and P9; Fig. 3). At last follow-up, four patients reported frequent use of inhaled β2 agonists, of which P5 and P8 require daily treatment. Routine spirometric testing showed persistent light or moderate airway obstruction in P3, P6, and P8 (Fig. 3). P8 reports limited physical capacities due to frequent respiratory infections with the remainder reporting no constraints.

## Discussion

The IEI resulting from bi-allelic deficiency of DOCK8 presents with combined T- and B-cell immunodeficiency. Hallmarks include frequent respiratory and skin infections, food allergies, eczema, eosinophilia, and elevated IgE. Subsequent organ damage, susceptibility to malignancies, and vascular complications entail high mortality and morbidity, justifying HSCT as a curative treatment option. Limited data on long-term outcomes for patients with DOCK8 deficiency and mixed chimerism post-HSCT are available, warranting this report on nine patients with a median follow-up of 78 months (total 727 patient months) post-HSCT and in-depth analysis of cell lineage-specific chimerism. Overall, we report successful HSCT after reduced toxicity conditioning in all patients with improvement of susceptibility to infections and allergies and resolution of eczema. Specifically, patients with a mixed chimerism present nearly complete T-cell chimerism, suggesting a selective advantage for wild-type donor T-cells, but lower donor B-cell chimerism with a possible tendency to lower IgG values and immunoglobulin substitution. No increased allergic, respiratory, or infectious complications were observed in patients with mixed chimerism. Secondary thyroid cancer in P2 was attributed to TBI conditioning and was not interpreted as an increased risk for oncogenesis in the context of mixed chimerism.

Aydin et al. reported on a large cohort of 81 patients with DOCK8 deficiency and HSCT on behalf of the inborn errors working party of EBMT and ESID with promising overall outcomes of HSCT [23]. More recently, Haskologlu et al. reported on a Turkish cohort of 20 patients with DOCK8 deficiency of whom 11 patients underwent HSCT [28]. However, these and other reports have focused on survival, conditioning regimens, and transplant-related outcomes, rather than improvement of clinical aspects and immunologic reconstitution, particularly in patients with mixed chimerism. Al-Herz et al. reported a retrospective review of 11 patients with ameliorated infectious and atopic symptoms post-HSCT, including an analysis of mixed lineage-specific chimerism in three patients, albeit with significantly shorter follow-up than our cohort [19]. Overall survival was 100% in our study, compared to 91% (Haskologlu et al.), 91% (Al-Herz et al.), and 84% (Aydin et al.), and none of our patients suffered from cGvHD. In patients with mixed chimerism, lineage-specific analysis showed a median of 97% (87–99%) of donor T-cells and 41% (30–85%) of donor B-cells, comparable to results from Al-Herz et al. (donor T-cells 82–97%, donor B-cells 0–46%, donor myeloid 0–7%) [19]. In line with our findings, Al-Herz et al. conclude that donor-derived T cells have a selective advantage. The role of DOCK8 in T-cell survival may provide a selective advantage for DOCK8 wild-type donor T-cells after HSCT, resulting in more complete T-cell donor chimerism [30, 31]. The extensive lymphocyte phenotype of DOCK8 deficiency and its correction post-HSCT have been catalogued by Pillay et al., although only in patients with complete donor T-cell chimerism [29]. Due to the retrospective nature of our study and lacking consent for biobanking, no further T-cell phenotyping or cytokine profiles are available for our patients for further assessment of T-cell reconstitution in DOCK8 deficiency with mixed chimerism. P2–P5 present with near complete T-cell chimerism with proper overall T-cell subset reconstitution, and therefore, we expect comparable T-cell phenotypes to patients with complete chimerism as shown by Pillay et al. [29]. P1, however, with 87% CD3^+^ donor T-cells has reduced thymopoiesis after partial thymectomy, weakening overall interpretation of T-cell subsets for patients with mixed chimerism. Further analysis of T-cell subsets for P1 would therefore not add meaningful knowledge to T-cell reconstitution in mixed chimerism for DOCK8 deficiency. Therefore, we believe our cohort may add additional information for T-cell reconstitution post-HSCT in patients with mixed chimerism.

Other studies found lower proliferative rates of DOCK8-deficient B-cells [8, 11]. Conversely, we observed no relevant selective advantage for wild-type B-cells in vivo as evidenced by an overall lower but stable B-cell chimerism over time after HSCT, but subtle effects cannot be excluded due to the low number of patients. Myeloid chimerism was not available for our patients, but a selective advantage for wild-type DOCK8 in lymphoid cells, rather than myeloid cells, was previously shown [32].

DOCK8 deficiency has been shown to impair immunological synapses through ICAM-1 in mouse models and reduced persistence in germinal centers and affinity maturation or differentiation into marginal zone cells in DOCK8-deficient B-cells [33]. Additionally, decreased generation of memory B-cells with diminished long-lasting antibody production has been related to impaired B-cell signaling [2, 11] and reduced B-cell proliferation was seen in patients with DOCK8 deficiency [11, 29]. DOCK8 deficiency has been shown to disrupt B-cell responses to signals via TLR, BCR, CD40, and cytokines, especially IL-21 [29]. In one patient, Al-Herz et al. found increased donor chimerism in the switched memory B-cell compartment, in agreement with recent findings of DOCK8 enrichment in the memory B-cells [32]. However, we did not analyze this in our patients but reduced switched memory B-cell numbers were not clearly associated with mixed chimerism in our cohort. The latter is exemplified by P5 with hypogammaglobulinemia and a higher CD19^+^ donor B-cell chimerism, but the lowest CD19^+^ B-cell count and reduced memory B-cells as compared to other patients with mixed chimerism. B-cell reconstitution post-HSCT is variable and influenced by many factors in pediatric patients [34]. Additionally, DOCK8-mutant B cells are unable to form marginal zone B cells or to persist in germinal centers and to undergo affinity maturation [33]. Split chimerism, with allogeneic T-cells but persistent autologous B-cells, impairs reconstitution of humoral immunity and has been implied with defective IL-21 receptor signaling in T-cell-dependent B-cell differentiation post-HSCT in patients with X-SCID and JAK3-SCID [35]. Our data show a possible tendency to hypogammaglobulinemia and necessity for immunoglobulin substitution in patients with mixed chimerism, but with the limited data available, no causal claim for DOCK8-specific effects can be made. Further research is required to elucidate the effect of split chimerism on B-cell function and adaptive humoral immunity reconstitution in general and particularly in DOCK8 deficiency.

We observed no correlation between persistent airway obstruction post-HSCT and mixed chimerism. Persistent food allergies post-HSCT are commonly seen in DOCK8-deficient patients without correlation with chimerism and are slow to improve post-HSCT [12], presumably due to persistence of long-lived IgE-producing plasma cells [36] and tissue-resident memory B-cells. Happel et al. report similar results for 12 patients and detected allergen-specific mast cells through skin prick tests after HSCT [36]. Only few of our patients reported challenging prior food allergies post-HSCT; however, reintroduction of a diversified diet post-HSCT has been successfully reported for numerous other patients [28, 36–38]. The low incidence of allergic events post-HSCT, despite overall stable allergen reactivity on immunoblot, may be attributed to higher T-cell chimerism and a speculative role of regulatory T-cell (T_reg_) functions. Janssen et al. demonstrated reduced suppressive activity of T_reg_ in DOCK8 deficiency [39]. T_reg_ activity may contribute to improvement of eczema in our patients and previous reports [23, 37, 38]. Unfortunately, no evaluation of T_reg_ was available for our patients. A possible role for T_reg_ suppression of T_H_2-cells in atopic dermatitis is reviewed by Agrawal et al. [40]. Interestingly, T_reg_ express an affinity towards skin-homing through CCR4, CCR6, and cutaneous lymphocyte-associated antigen (CLA), a similar mechanism as seen in IPEX syndrome, caused by a T_reg_ defect [40]. Further studies are necessary to elucidate the role of T_reg_ in DOCK8 deficiency. Unfortunately, the importance of NK-cell immunity in defense against virus infections cannot be properly addressed by this report, due to missing appropriate functional assays [14].

We believe that early diagnosis and timely curative management are crucial in DOCK8 deficiency as non-reversible organ damage may occur due to infectious or auto-inflammatory complications, but may be delayed by varying phenotypes or near-normal DOCK8 expression caused by hypomorphic variants or somatic reversions [5]. Somatic reversions were present in two of our patients and both present with complete donor chimerism post-HSCT. The importance of early detection is exemplified by P8, who was diagnosed at a later age than her sister P3 and suffers from structural pre-HSCT lung damage with frequent respiratory infections despite complete donor chimerism. In comparison, P3 presents as a healthy and active young girl, despite mixed chimerism. Somatic reversion in general may ameliorate disease course, but patients still do experience fatal complications [8]. Rare constellations such as somatic reversion of a hypomorphic DOCK8 allele leading to an atypical and relatively milder phenotype have been reported [9] and Pillay et al. recently reported in-depth analysis of partial somatic reversion in three patients with compound heterozygous mutations in *DOCK8* with clinical improvement over time [32]. Overall, T-cells showed highest levels of somatic reversion, in agreement with our findings regarding selective advantages for donor T-cells post-HSCT. Therefore, indication for HSCT in patients with somatic mosaicism should be evaluated individually, but we currently recommend HSCT with reduced conditioning for those with infectious complications to prevent organ damage and fatal complications. Hypothetically there could be long-term complications of clonal selection by somatic reversion, but Pillay et al. have not observed that [32]. Metabolomic biomarkers may support clinicians in future differential diagnosis of atopic dermatitis in order to achieve early diagnosis of DOCK8 deficiency [41], but currently, the diagnosis demands experienced clinicians and intracellular flow cytometry for DOCK8, most successfully detected in B-cells, which show minimal reversion [8], and confirmation by Sanger and/or exome sequencing.

With this report, we hope to increase our understanding of long-term immunologic outcomes post-HSCT for DOCK8 deficiency and support clinicians, patients, and families facing this debilitating disease. We highlight the importance of intracellular flow cytometry for DOCK8 for diagnosis and chimerism monitoring at the single cell level. A review of conditioning regimens is beyond the scope of this report, but overall, promising outcomes for reduced toxicity conditioning reported here and elsewhere [23] support the notion that such regimens are preferable for this disease, despite a higher frequency of mixed chimerism. Aware of the relatively small cohort analyzed, we could not demonstrate a consistent detrimental effect of mixed chimerism on immunological and clinical outcomes, especially the frequency of infections. Still, further research is needed to investigate the effect of mixed chimerism in DOCK8 deficiency, especially on cell lineages not covered in this report. Given this uncertainty, we advocate aiming for complete donor chimerism in treating DOCK8 deficiency, but suggest aiming at reduced toxicity over myeloablation, especially for patients with pre-existing organ damage.

## Supplementary Information

Below is the link to the electronic supplementary material.

**Figure S1: Immunologic reconstitution**. Kinetics of cell counts for leucocytes, lymphocytes, and lymphocyte subsets (CD3^+^, CD4^+^, CD8^+^, CD19_+_, CD16CD56^+^) are shown in months post-HSCT. Grey background denotes patients with mixed chimerism. Horizontal dashed lines define normal ranges. (PDF 45 KB)

**Figure S2: Course of IgG-values**. Course of serum IgG (g/l) pre- (time = 0) and post-HSCT. (PDF 27 KB)

## Data Availability

The datasets generated during and/or analyzed during the current study are available from the corresponding author on reasonable request. All data generated or analyzed during this study are included in this published article (and its supplementary information files).
