# Supplementary figures and images for: Lineage-Specific Chimerism and Outcome After Hematopoietic Stem Cell Transplantation for DOCK8 Deficiency
Source: J Clin Immunol. 2021 Jun 2;41(7):1536–48. doi: 10.1007/s10875-021-01069-5 (PMC8452590; doi:10.1007/s10875-021-01069-5)

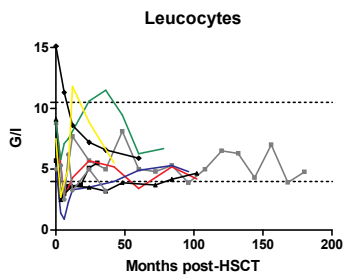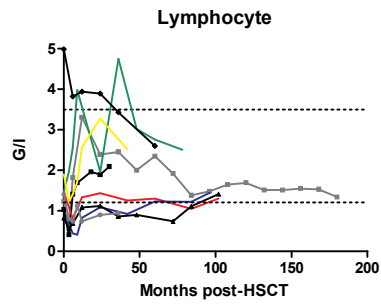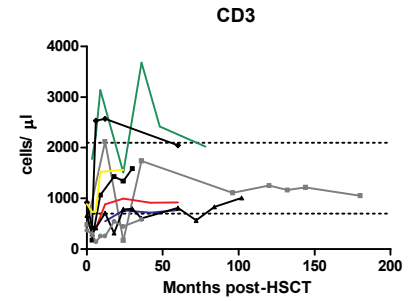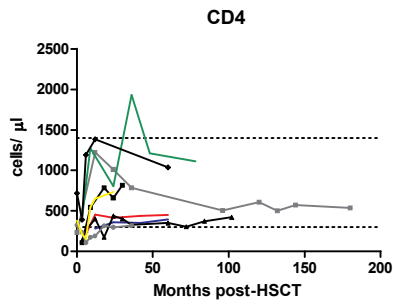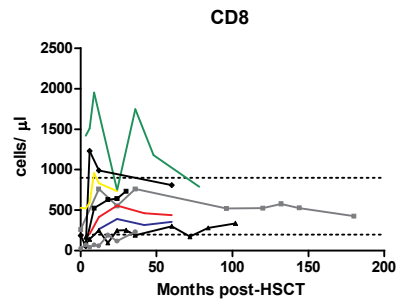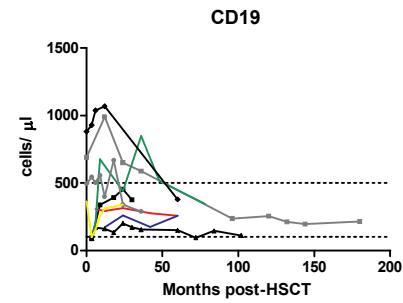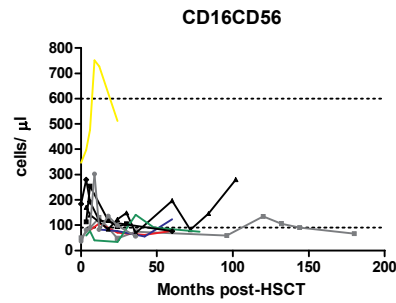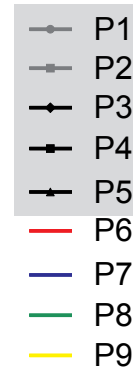

Supplement: Supplementary file 1 — Figure S1: Immunologic reconstitution. Kinetics of cell counts for leucocytes, lymphocytes, and lymphocyte subsets (CD3+, CD4+, CD8+, CD19+, CD16CD56+) are shown in months post-HSCT. Grey background denotes patients with mixed chimerism. Horizontal dashed lines define normal ranges. (PDF 45 KB) [file 10875_2021_1069_MOESM1_ESM.pdf]

# IgG

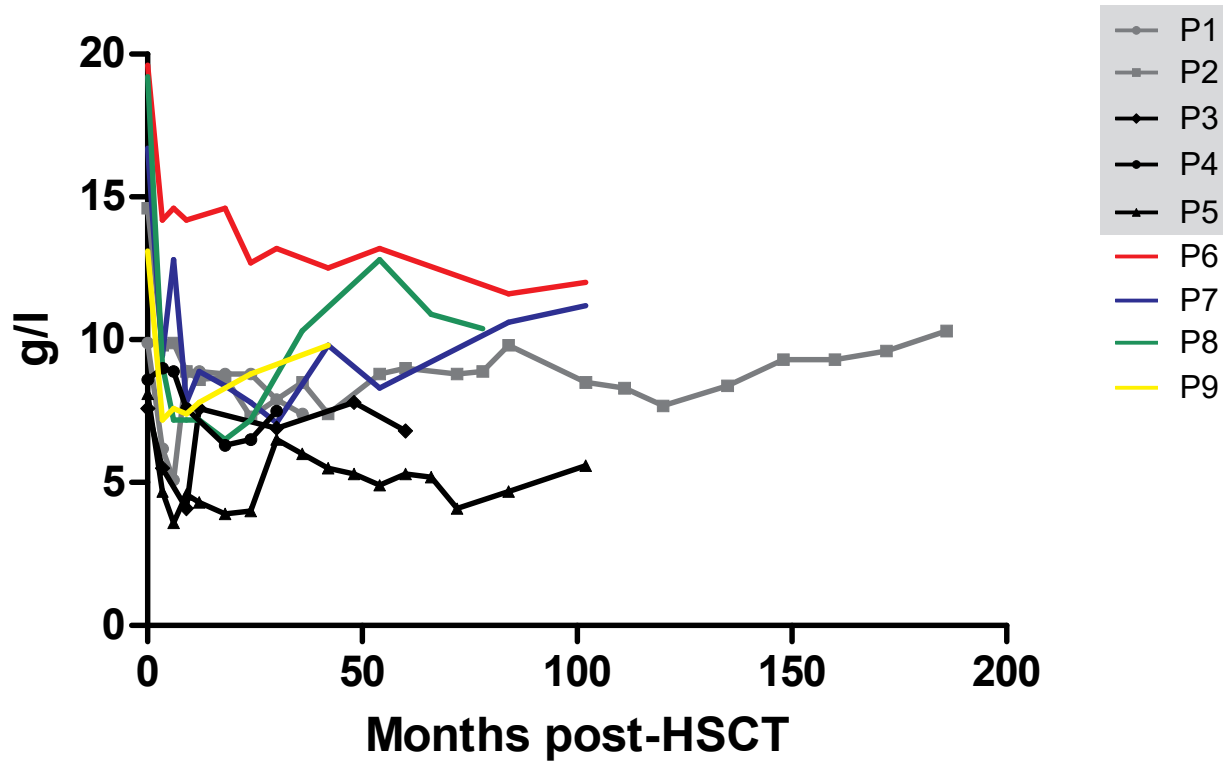

Supplement: Supplementary file 2 — Figure S2: Course of IgG-values. Course of serum IgG (g/l) pre- (time = 0) and post-HSCT. (PDF 27 KB) [file 10875_2021_1069_MOESM2_ESM.pdf]
